# Quality of life and subjective sleep-related measures in bipolar disorder and major depressive disorder

**DOI:** 10.1007/s11136-021-02929-8

**Published:** 2021-07-14

**Authors:** Françoise Jermann, Nader Perroud, Sophie Favre, Jean-Michel Aubry, Hélène Richard-Lepouriel

**Affiliations:** Geneva University Hospitals, Geneva, Switzerland

**Keywords:** Quality of life, Sleep, Bipolar, Major depressive disorders

## Abstract

**Purpose:**

Diminished quality of life (QoL) has been reported in patients with mood disorders. QoL has also been shown to be decreased by sleep disturbances. Since sleep disorders are common in mood disorders, the aim of this study was to determine whether sleep characteristics are associated to QoL among patients with Bipolar Disorder (BD) and unipolar Major Depressive Disorders (MDD).

**Methods:**

QoL was assessed in 170 patients with mood disorders (61 BD and 109 MDD), who also completed questionnaires measuring the severity of insomnia, sleepiness, chronotype preference and obstructive sleep apnea (OSA) probability index.

**Results:**

Analyses showed that BD and MDD groups had similar QoL and sleep measures but the MDD group had higher OSA scores. In BD, correlations indicated a relationship between QoL and insomnia complaints and sleepiness, whereas in MDD, correlations indicated an association between QoL and insomnia complaints and OSA score. In both groups, QoL was related to depressive symptomatology. Linear regressions showed that, in BD, QoL was related to insomnia complaints and sleepiness even in the euthymic state, whereas in MDD, QoL was related to insomnia complaints but not in euthymic patients.

**Conclusion:**

QoL is related to sleep differently in BD and MDD. The results suggest that insomnia and sleepiness are particularly high in BD even when patients are euthymic. These findings suggest that focusing on insomnia and sleepiness during different mood states of BD could increase QoL.

## Introduction

Patients with bipolar disorders (BD) and major depressive disorders (MDD) report low quality of life (QoL) [1, 2]. There is no consensual definition of QoL in mood disorders but a recent thematic analyses in the domain of BD highlighted two dimensions: on the one hand, the evaluation of subjective experience, satisfaction, and well-being, and on the other hand, the observation of deviations from the standard in various domains of functioning (e.g., physical, mental, emotional, social, occupational, health) [3]. In this study, QoL corresponds to the satisfaction with life in specific domains that are central in BD. These areas are related to clinical features (mood, sleep, health, cognition), pragmatic and functional outcomes (household, work, leisure, finances) and personal or social-orientated constructs (social, self-esteem, spirituality, identity, independence). In mood disorders, poor QoL is correlated to depression severity but changes in mood symptomatology do not totally account for the variance in QoL [4] and poor QoL persists even in euthymic BD patients compared to controls [5].

Independently of mood disorders, poor QoL has also been shown in people presenting sleep disruptions, such as insomnia [6], sleepiness [7], obstructive sleep apnea (OSA) [8], and in people who have a preference for an evening chronotype [9]. These sleep particularities are common in both BD and MDD [10]. In BD patients, insomnia or hypersomnia is common during the depressive phase and continues even in the euthymic phase [11]. Moreover, it has been demonstrated that insomnia is a prodrome to both depressive onset and manic onset and is associated with more severe illness [12]. Sleepiness (decreased vigilance) has been described as excessive during depressive states in BD patients [13]. Patients with BD also have a preference for an evening chronotype [14] and have a high prevalence of OSA (24.5%).

Finally, regarding specifically the relation between QoL and sleep parameters in BD patients, it has been shown that sleep alteration (e.g., complaints, dissatisfaction with sleep, biological rhythm disruption) contributes to low QoL in symptomatic [15, 16] and euthymic BD patients [17]. Moreover, BD patients with abnormal sleep duration (short sleepers < 6 h or long sleepers > 9 h or > 10 h) reported poorer QoL than BD patients with normal sleep duration [18, 19]. This has however not been shown in euthymic BD patients [17].

MDD patients show similar sleep characteristics to BD patients. Indeed, insomnia is a frequent sleep disturbance during MDD and persists even after remission [20, 21]. Sleepiness [22] and evening chronotype [22] are also common in MDD and the prevalence of OSA (36.6%) is even higher than that in BD patients [23].

In summary, QoL is low in people with mood disorders as well as those with sleep disturbances. The present study aimed to explore within a single trial the relation between QoL and sleep characteristics (insomnia complaints, sleepiness, chronotype, and risk for OSA) among individuals with BD or MDD.

## Method

### Population

One hundred and seventy participants were recruited from the outpatient Mood Disorder Unit at the Geneva University Hospitals. Each patient was assessed during three sessions by a psychiatrist and a psychologist specializing in adult mood disorders. Diagnoses were established according to the ICD-10 (International Classification of Diseases-10) criteria [10] and confirmed by the MINI (Mini-International Neuropsychiatric Interview)[24]. Current severity of depressive symptomatology was assessed with the Montgomery-Asberg Depressive rating scale (MADRS) [25] and hypo/manic symptoms with the Young Mania Rating Scale (YMRS) [26]. The assessments were carried out in French.

This study was approved by the ethics committee of the Geneva University Hospitals and informed written consent was obtained from all participants.

### Questionnaires

Brief Quality of Life (Brief QoL.BD) [27]; French version: [28]). The QoL.BD and its short version (Brief QoL.BD) were developed to measure QoL in BD. The authors assumed that some symptoms of BD could have a unique impact upon QoL and could be more sensitive to change than generic QoL measures. The QoL.BD and the Brief QoL.BD measure the subjective evaluation of well-being and the functioning dimension of QoL and was developed to be used in all BD mood states (depressive, hypomanic, euthymic). Twelve aspects of QoL—physical, sleep, mood, cognition, leisure, social, spirituality, finances, household, self-esteem, independence and identity—are rated on a five-point Likert scale (range 1–5). The total score ranges from 12 to 60, with higher scores representing better QoL.

Insomnia Severity Index (ISI) [29] French version: [30]). The ISI comprises seven items that are scored on a five-point Likert scale (range 0–4). The total score ranges from 0 to 28, with higher scores indicating more insomnia complaints.

Epworth Sleepiness Scale (ESS) [31] French version: [32]. The ESS is composed of eight questions that are scored on a four-point Likert scale (range 0–3) indicating the probability of falling asleep in different situations in daily life. The final score ranges from 0 to 24. A score above 10 is considered to indicate excessive sleepiness.

Reduced version of the Horne and Östberg Morningness—Eveningness Questionnaire (rMEQ) [33] French version: [34]. The rMEQ is composed of five questions. The total score ranges from 4 to 25, with a lower score indicating an evening preference and a higher score a morning preference.

STOP-Bang questionnaire [35] French version: [36]. The STOP-Bang questionnaire is composed of eight items assessing the risk factors for obstructive sleep apnea. Questions are scored on a yes/no scale. Each positive answer corresponds to one point (range 0–8). A higher score corresponds to a higher risk for OSA. A score of three or more indicates a moderate risk for OSA.

### Statistical analyses

Statistics were computed using SPSS version 25 (IBM, Armonk, NY). QoL and variables related to sleep were all normally distributed (all skewness values were between − 0.20 and 0.87 and kurtosis values were between − 0.89 and 0.46). Differences between groups for demographic and clinical variables were tested with Student’s *t*-tests and chi-square tests as appropriate. Associations between QoL, sleep measures and mood symptoms were tested with Pearson correlation coefficients. Multiple linear regressions were performed with QoL as dependent variable and sleep measures (insomnia symptoms, sleepiness, morningness-eveningness and risk for OSA) as independent variables. Analyses were performed separately for the BP and MDD groups. The same multiple linear regression analyses were then done on a subsample composed of only euthymic patients, defined with a MADRS score < 11 and a YMRS score < 6. Significance level was set at *p* < 0.05.

## Results

Demographic, clinical and psychometric data are presented in Table 1. There are no significant differences between patients with BD and MDD for demographic data and for QoL and sleep measures (insomnia, sleepiness, morningness-eveningness) except for the risk for OSA and BMI, which are higher in the MDD than the BD group (Table 1). For each group, the type of psychotropic drugs taken are presented in Table 1 (15% of the participants in the BD group and 24% in the MDD group didn’t take any psychotropic drug—*X*^2^ = 1.98;* p* = 0.16). Given the variety of molecules and dosage, this information is descriptive and could not be included in the analyses.

**Table 1:** Demographic and clinical characteristics and psychometric data (mean-SD) for patients with BD and MDD

	BD (*n* = 61)	MDD (*n* = 109)	*t*/*X*^2^	d*f*	*p*
	BD-I 23%				
	BD-II 74%				
	Cyclothymia 3%				
Age	43.03 (12.95)	45.06 (13.01)	0.97	168	.33
Female	75%	63%	2.62	1	.11
Education (highest level)			3.67	3	.30
- Elementary school	10%	12%			
- Apprenticeship training	29%	39%			
- High school	15%	18%			
- University or similar	46%	31%			
Civil status			1.15	2	.56
- Single	29%	38%			
- Married or living as married	61%	53%			
- Divorced, separated or widowed	10%	9%			
Body Mass Index (BMI)	24.38 (5.55)	26.50 (5.56)	2.36	165	< .05
Comorbidity					
- Mental and behavioral disorders due to psychoactive substance use	16%	16%	0.02	1	.90
- Schizophrenia, schizotypal and delusional disorders	3%	0%	3.62	1	.06
- Neurotic, stress-related and somatoform disorders	18%	28%	1.93	1	.17
- Behavioral syndromes associated with physiological disturbances and physical factors	3%	2%	0.36	1	.55
- Disorders of adult personality and behavior	13%	7%	1.53	1	.22
- Attention-Deficit Hyperactivity Disorder	2%	5%	0.99	1	.32
Psychotropic drugs					
- Antidepressants	38%	65%			
- Antiepileptics	25%	13%			
- Antipsychotics	49%	22%			
- Mood stabilizers	20%	4%			
- Benzodiazepine	30%	28%			
- Hypnotics	20%	18%			
- Stimulants	–	1%			
Questionnaires					
- Depressive symptomatology (MADRS)	12.86 (9.85)	15.84 (10.44)	1.76	156	.08
- Manic symptomatology (YMRS)	1.16 (2.09)	0.72 (1.76)	1.40	155	.17
- Quality of life (Brief QoL.BD)	36.67 (10.22)	35.77 (10.64)	0.53	168	.59
- Insomnia severity index (ISI)	10.73 (6.94)	12.88 (6.99)	1.91	165	.06
- Epworth sleepiness scale (ESS)	8.59 (4.45)	8.35 (5.43)	0.30	167	.77
- Reduced morningness-eveningness questionnaire (rMEQ)	14.90 (3.88)	14.01 (4.14)	1.37	166	.17
- Risk for obstructive sleep apnea (STOP-Bang)	1.85 (1.60)	2.43 (1.52)	2.31	164	< .05

Pearson correlations between QoL and sleep measures are displayed in Table 2. Among patients with BD, there are significant relations between QoL and insomnia complaints and sleepiness. In the MDD group, QoL was negatively correlated with insomnia complaints and the risk for OSA. In both groups, QoL was negatively associated with depressive symptomatology. Depressive symptomatology was also positively correlated with insomnia symptoms (both groups) and the risk for OSA (MDD group). There was, however, no significant correlation between QoL and age for the BD (r = − 0.01; *p* = 0.94) or MDD groups (r = − 0.03; *p* = 0.78) and no significant difference in QoL score across gender in either group (BD: t(59) = 0.78; *p* = 44; MDD: t(107) =  − 1.19; *p* = 0.24).

**Table 2:** Pearson correlations between QoL, sleep measures and mood symptoms and multiple linear regressions with QoL as dependent variable for BD and MDD

	Correlations					Multiple linear regressions			
	Brief QoL.BD	ISI	ESS	rMEQ	STOP-Bang	Beta	*p*	95% CI	
								Lower	Upper
BD									
ISI	− .45**	–				− .05	.000	− 1.08	− 0.40
ESS	− .27*	− .15	–			− .33	.008	− 1.31	− 0.21
rMEQ	.10	− .22	.02	–		− .01	.96	− 0.62	0.58
STOP–Bang	− .18	.07	.36**	− .09	–	− .03	.81	− 1.71	1.35
MADRS	− .58**	.31*	.07	− .11	.06				
YMRS	.24	.07	− .20	.05	− .04				
MDD									
ISI	− .31**	–				− .32	.001	− 0.77	− 0.20
ESS	− .11	.16	–			− .13	.17	− 0.61	0.11
rMEQ	.02	− .02	− .07	–		.03	.74	− 0.38	0.53
STOP–Bang	− .20*	.17	.23*	.11	–	− .14	.17	− 2.29	0.41
MADRS	− .64**	.34**	− .08	.01	.24*				
YMRS	.17	.15	.06	.07	.02				

*Brief QoL.BD* quality of life; ISI: insomnia severity index, *ESS* epworth sleepiness scale, *rMEQ* reduced version of the Horne and Ostberg Morningness-Eveningness Questionnaire, *STOP-Bang* risk factors for obstructive sleep apnea – STOP-Bang questionnaire, *MADRS* montgomery-asberg depression rating scale, *YMRS* young mania rating scale, * < .05; ** < .01; 95% *CI* 95% confidence interval

To evaluate whether sleep variables are related to QoL, multiple linear regressions were conducted for both groups. Underlying assumptions required for regressions analyses were all met (linearity, multicollinearity, normality of residuals, and homoscedasticity of residuals). In the BD group, the lowest tolerance value was 0.84 and the highest VIF value was 1.19, Durbin-Watson = 1.56, maximum Cook’s distance value was 0.11. In the MDD group, the lowest tolerance value was 0.90 and the highest VIF value was 1.12, Durbin-Watson = 1.81, maximum Cook’s distance value was 0.08. In patients with BD, QoL was explained by insomnia complaints and sleepiness. In MDD, QoL was related only to insomnia complaints (Table 2).

To rule out the impact of actual depressive or hypo/manic symptomatology, participants with BD and MDD in a euthymic state were compared (Table 3). Student’s *t*-tests showed that euthymic patients with BD and MDD did not differ neither regarding QoL nor for the sleep variables (see Table 3). Multiple linear regressions were conducted with QoL as dependent variable and sleep measures as predictors; the results indicated that, for euthymic patients with BD, QoL was related to insomnia complaints and sleepiness, whereas for euthymic patients with MDD, sleep parameters did not affect QoL (Table 3, 4).

**Table 3:** Demographic characteristics, psychometric data (mean-SD) for the euthymic subsample of patients with BD and MDD

	Euthymic BD (*n* = 25)	Euthymic MDD (*n* = 32)	*t*/*X*^2^	d*f*	*p*
Age	44.52 (10.89)	42.78 (11.79)	0.57	55	.57
Female	68%	66%	0.04	1	.85
Education (highest level)			0.76	3	.86
- Elementary school	12%	6%			
- Apprenticeship training	40%	38%			
- High school	12%	12%			
- University or similar	36%	44%			
Civil status			0.64	2	.73
- Single	28%	25%			
- Married or living as married	68%	66%			
- Divorced, separated or widowed	4%	9%			
Questionnaires					
- Quality of life (Brief QoL.BD)	41.08 (9.11)	43.66 (8.84)	− 1.08	55	.29
- Insomnia severity index (ISI)	9.50 (6.36)	9.60 (6.22)	− 0.06	55	.95
- Epworth sleepiness scale (ESS)	9.56 (4.70)	8.16 (5.10)	1.07	55	.29
- Reduced morningness-eveningness questionnaire (rMEQ)	15.32 (3.92)	14.23 (3.85)	1.05	55	.30
- Risk for obstructive sleep apnea (STOP-Bang)	2.08 (1.78)	2.03 (1.65)	1.01	53	.92

**Table 4:** Multiple linear regressions with QoL as dependent variable for the euthymic subsample of patients with BD and MDD

Multiple linear regressions	Beta	*p*	95% CI	
			Lower	Upper
BD				
ISI	− .52	.005	− 1.24	− 0.25
ESS	− .53	.007	− 1.74	− 0.32
rMEQ	.004	.98	− 0.79	0.81
STOP-Bang	.28	.12	− 0.39	3.29
MDD				
ISI	− .30	.16	− 0.92	0.16
ESS	.03	.88	− 0.63	0.73
rMEQ	.001	.99	− 0.79	0.80
STOP-Bang	− .11	.61	− 2.65	1.59

*Brief QoL.BD* Quality of life, *ISI* Insomnia Severity Index, *ESS*, Epworth Sleepiness Scale, *rMEQ* reduced version of the Horne and Ostberg Morningness-Eveningness Questionnaire, *STOP-Bang* risk factors for obstructive sleep apnea—STOP-Bang questionnaire, * < .05; ** < .01; 95% *CI* 95% confidence interval

## Discussion

This study investigated the relationship of QoL and sleep in patients with mood disorders. The patients with BD and MDD reported similar QoL and sleep characteristics (insomnia complaints, sleepiness, morningness-eveningness), although the MDD group displayed a higher risk for OSA than the BD group. There were significant negative correlations between QoL and depressive symptomatology in both groups, as well as between QoL and insomnia and sleepiness in BD and QoL, and insomnia and risk for OSA in MDD. Finally, the results showed that, in patients with BD, QoL was related to insomnia complaints and sleepiness level, whereas in MDD patients only insomnia complaints were associated to QoL. Considering only euthymic patients, insomnia complaints and sleepiness were still related to QoL in BD, whereas in MDD, QoL was not associated to any sleep parameters.

In patients with BD, QoL is related to insomnia complaints and sleepiness even in the euthymic state. These results are particularly interesting as they bring together research showing, on the one hand, that QoL is associated to insomnia or sleepiness [6, 7] and, on the other hand, that patients with BD complain of insomnia and sleepiness [11, 13]. Moreover, the results are also congruent with prior studies showing that altered sleep parameters are related to low QoL in BD patients [15, 16, 18, 19]. The relationship between QoL and sleepiness is especially interesting as it could be related to some of the pharmacological treatments used to treat BD (e.g., antipsychotics) but also to possible impairment in daily functioning in BD (e.g., impaired occupational, cognitive, interpersonal functioning) [37]. Further studies should be conducted to specifically evaluate the relationship between QoL, sleepiness, the use of sedatives and functional impairment.

The analyses showing a relationship between QoL and insomnia in BD are an invitation to consider specific interventions to reduce insomnia and sleepiness during different stages of the disorder (depressive and euthymic). To date, some studies evaluated Cognitive Behavioral Therapy for Insomnia (CBT-I) among BD patients and showed positive effects on reducing insomnia [e.g., 38, 39, 40]. However, data regarding CBT-I in BD are still scarce probably because the intervention requires spending less time in bed; this is sometimes interpreted as shortening sleep duration, which is known to be a risk factor for the onset of hypo/manic episodes [41]. Today, the recommendation for BD patients who undergo CBT-I is that depressive and hypo/manic symptoms are monitored at every session and sleep restriction suspended in case of symptom change [42, 43].

Considering patients with MDD, this study shows that QoL is related to insomnia and the risk for OSA. These results are in accordance with past findings demonstrating both that insomnia and OSA are frequent in MDD [21, 23] and that insomnia and OSA are related to decreased QoL [6, 8]. Moreover, these results are an important reminder for clinicians to systematically screen MDD patients for the risk for OSA, given that it is associated with perturbed sleep and impaired physical health (e.g., obesity, cardiovascular diseases). It is also an invitation to be particularly careful about the pharmacological treatments used for MDD, as side effects include weight gain, which is a factor contributing to the risk for OSA. Our results showed that QoL is only related to insomnia in a sample including a whole range of depressive symptoms but not when patients with MDD are in a euthymic state; this suggests that the relation is mood-dependent. In other words, QoL seems to be related to insomnia when there is current depressive symptomatology.

### Limitations

This study has several strengths: a relatively large sample of thoroughly diagnosed patients with mood disorders and a variety of sleep characteristic measures. Still, some limitations must be acknowledged. First, even though the data are based on validated self-report questionnaires, measuring sleep parameters with objective tools (e.g., wrist actigraphy) or daily sleep diaries could have added further information. Second, the majority of the sample was taking medications, which could have affected their sleep and/or arousal level. Given the diversity of molecules and dosage among the sample, this dimension could not be taken into account in the analyses. However, the use of medication might be a mediator between QoL and sleep characteristics and then affect differently the relation for BD and MDD. Therefore, further studies should take this variable into account. Third, QoL was measured with a questionnaire specifically developed to assess QoL in BD whereas the sample also included patients with MDD. However, the data indicated that internal consistencies were similar in both groups (Cronbach’s alpha of 0.88 and 0.89, respectively) and there was also no difference between groups at an item level (all ps > 0.13).

## Conclusion

The results of this study suggest that in BD there is a link between QoL and insomnia complaints and sleepiness during the acute and euthymic phases of the disorder. These results have important implications for clinical practice as they encourage clinicians to evaluate insomnia complaints and sleepiness but also to suggest specific interventions for insomnia and sleepiness, not only during the acute phase of the disorder but also in the euthymic phase.
